# Whole genome single nucleotide polymorphism based phylogeny of *Francisella tularensis *and its application to the development of a strain typing assay

**DOI:** 10.1186/1471-2180-9-213

**Published:** 2009-10-07

**Authors:** Gagan A Pandya, Michael H Holmes, Jeannine M Petersen, Sonal Pradhan, Svetlana A Karamycheva, Mark J Wolcott, Claudia Molins, Marcus Jones, Martin E Schriefer, Robert D Fleischmann, Scott N Peterson

**Affiliations:** 1 Pathogen Functional Genomics Resource Center, J. Craig Venter Institute, Rockville, MD 20850, USA; 2 National Center for Zoonotic, Vector-Borne and Enteric Diseases, Division of Vector-Borne Diseases, CDC, Fort Collins, CO, 80521, USA; 3 USAMRIID, Fort Detrick, MD, 21702, USA

## Abstract

**Background:**

A low genetic diversity in *Francisella tularensis *has been documented. Current DNA based genotyping methods for typing *F. tularensis *offer a limited and varying degree of subspecies, clade and strain level discrimination power. Whole genome sequencing is the most accurate and reliable method to identify, type and determine phylogenetic relationships among strains of a species. However, lower cost typing schemes are necessary in order to enable typing of hundreds or even thousands of isolates.

**Results:**

We have generated a high-resolution phylogenetic tree from 40 *Francisella *isolates, including 13 *F. tularensis *subspecies *holarctica *(type B) strains, 26 *F. tularensis *subsp. *tularensis *(type A) strains and a single *F. novicida *strain. The tree was generated from global multi-strain single nucleotide polymorphism (SNP) data collected using a set of six Affymetrix GeneChip^® ^resequencing arrays with the non-repetitive portion of LVS (type B) as the reference sequence complemented with unique sequences of SCHU S4 (type A). Global SNP based phylogenetic clustering was able to resolve all non-related strains. The phylogenetic tree was used to guide the selection of informative SNPs specific to major nodes in the tree for development of a genotyping assay for identification of *F. tularensis *subspecies and clades. We designed and validated an assay that uses these SNPs to accurately genotype 39 additional *F. tularensis *strains as type A (A1, A2, A1a or A1b) or type B (B1 or B2).

**Conclusion:**

Whole-genome SNP based clustering was shown to accurately identify SNPs for differentiation of *F. tularensis *subspecies and clades, emphasizing the potential power and utility of this methodology for selecting SNPs for typing of *F. tularensis *to the strain level. Additionally, whole genome sequence based SNP information gained from a representative population of strains may be used to perform evolutionary or phylogenetic comparisons of strains, or selection of unique strains for whole-genome sequencing projects.

## Background

*Francisella tularensis*, a Gram-negative bacterium, is the causative agent of tularemia and a Category A select agent. *F. tularensis *is divided into three subspecies (subsp.): *tularensis *(type A); *holarctica *(type B); and *mediasiatica *[1,2]. Tularemia caused by type A strains occurs only in North America, whereas tularemia caused by type B strains occurs throughout the northern hemisphere. Together these two species account for the majority of cases of tularemia worldwide. *F. tularensis *subsp. *mediasiatica *includes strains predominant in central Asia [3]. *F. novicida *has been suggested to be a subspecies of *F. tularensis *based on genetic similarity [4,5], but is still formally recognized as a distinct species. *F. novicida *has been isolated from North America and Australia, and rarely causes human disease even though it can cause a lethal infection in the murine model of disease [3,6].

Current DNA based genotyping methods for typing *F. tularensis *offer a varying degree of power to discriminate subspecies, clades and strains [2,7,8]. Two clades, A1 and A2, within *F. tularensis *subsp. *tularensis *have been reported based on multiple subtyping methods including multi-locus variable number tandem repeat analysis (MLVA), pulsed-field gel electrophoresis (PFGE) and multi-locus sequence typing [2]. Clades within A1, A1a and A1b, have been identified by PFGE [9]. A limited degree of variation has been observed within type B strains by all methods. MLVA currently provides the highest degree of strain discrimination for *F. tularensis*, however it is limited in its ability to perform evolutionary analyses and to estimate relationships among very closely related strains [10].

Development of high-resolution genotyping methods for *F. tularensis *can ideally be met by whole genome sequencing of multiple strains. Whole genome sequencing is the most accurate and reliable method to identify and discriminate strains of a species, especially those species with a high degree of genome homogeneity. Genomic sequence information of several type A and B strains is now available http://www.ncbi.nlm.nih.gov/sites/entrez?db=genomeprj&orig_db=&term=Francisella%20tularensis&cmd=Search. *F. tularensis *has a single circular chromosome with genome size of ~1.89 Mb. Naturally occurring plasmids have not been reported for *F. tularensis *strains so far. A low genetic diversity in *F. tularensis *has been documented. Based on whole genome sequencing, the genetic variation between the type B live vaccine strain (LVS) and two other type B strains, FSC200 and OSU18, is only 0.08% and 0.11% respectively. *F. tularensis *subsp. *holarctica *strain FSC200 is a virulent strain of European origin whereas *F. tularensis *subsp. *holarctica *strain OSU18 is a virulent strain isolated in the United States. A higher genetic variation of 0.7% has been reported between a type B (LVS) and type A (SCHU S4) strain [11]. Global single nucleotide polymorphism (SNP) information, based on whole genome sequencing, offers several advantages over existing typing methods because each individual nucleotide may be a useful genetic character. The cumulative differences in two or more sequences provide a larger number of discriminators that can be used to genotype and distinguish bacterial strains. Strain genotypes that are built upon SNP variation are highly amenable to evolutionary reconstruction and can be readily analyzed in a phylogenetic and population genetic context to: i) assign unknown strains into well-characterized clusters; ii) reveal closely related siblings of a particular strain; and iii) examine the prevalence of a specific allele in a population of closely related strains that may in turn correlate with phenotypic features of the infectious agent [12]. SNPs also provide potential markers for the purpose of strain identification important for forensic and epidemiological investigations.

Previously, we reported an Affymetrix GeneChip^® ^based approach for whole genome *F. tularensis *resequencing and global SNP determination [13]. We now report the whole genome sequence and global SNP data from 40 *Francisella *strains using this approach. Twenty six *F. tularensis *type A (20 A1 and 6 A2), thirteen *F. tularensis *type B and one *F. novicida *strain were used for phylogenetic SNP analysis and identification of high-quality SNPs for use as typing markers. Based on our global analysis of 40 genomes, we were able to identify a series of SNPs at various levels of hierarchy. We used these SNPs to develop and validate a low-cost PCR-based assay for typing and discriminating *F. tularensis *isolates.

## Methods

### *Francisella *strains

*Francisella *strains used for whole genome sequencing are listed in Table 1. Strains used for evaluation of diagnostic SNP markers are shown in Table 2. All strains were identified as either type A or type B by glycerol fermentation or PCR. Pulsed field gel electrophoresis using *Pme*I was performed for CDC strains to characterize type A strains as either A1, A2, A1a or A1b [14]. Ribotyping, using the Dupont Qualicon RiboPrinter and *Pvu*II restriction enzyme, was used to characterize USAMRIID type A strains as A1 or A2 (USAMRIID, unpublished method).

**Table 1:** *Francisella *strains resequenced in the study

**S. No**.	Isolate	Species/Subspecies	**Clade**^a^	Other strain name	Geographic Source	Year isolated	Source
1	SCHUS4	*F. tularensis *type A	A1 (A1a)		Ohio	1941	CDC

2	MA00-2987	*F. tularensis *type A	A1 (A1b)		Massachusetts	2000	CDC

3	AR01-1117	*F. tularensis *type A	A1 (A1b)		Arkansas	2001	CDC

4	KS00-1817	*F. tularensis *type A	A1 (A1a)		Kansas	2000	CDC

5	OK00-2732	*F. tularensis *type A	A1 (A1b)		Oklahoma	2000	CDC

6	FRAN005	*F. tularensis *type A	A1		Illinois	1990	USAMRIID

7	FRAN006	*F. tularensis *type A	A1		Illinois	1988	USAMRIID

8	FRAN007	*F. tularensis *type A	A1		Illinois	1988	USAMRIID

9	FRAN008	*F. tularensis *type A	A1		Illinois	1988	USAMRIID

10	FRAN009	*F. tularensis *type A	A1		Illinois	1988	USAMRIID

11	FRAN010	*F. tularensis *type A	A1		Illinois	1987	USAMRIID

12	FRAN011^b^	*F. tularensis *type A	A1		Illinois	1984	USAMRIID

13	FRAN014	*F. tularensis *type A	A1		Illinois	1989	USAMRIID

14	FRAN015	*F. tularensis *type A	A1		Illinois	1988	USAMRIID

15	FRAN023	*F. tularensis *type A	A1	FoxP1	Ohio	1940	USAMRIID

16	FRAN026	*F. tularensis *type A	A1	Schu-SOO	Unknown	Unknown	USAMRIID

17	FRAN030	*F. tularensis *type A	A1	SOL	Unknown	Unknown	USAMRIID

18	FRAN031	*F. tularensis *type A	A1	SCHERM	Ohio	1944	USAMRIID

19	FRAN032	*F. tularensis *type A	A1	GREU	Ohio	Unknown	USAMRIID

20	FRAN033	*F. tularensis *type A	A1	HUGH	Ohio	1940	USAMRIID

21	WY96-3418	*F. tularensis *type A	A2		Wyoming	1996	CDC

22	CA02-0099	*F. tularensis *type A	A2		California	2002	CDC

23	UT02-1927	*F. tularensis *type A	A2		Utah	2002	CDC

24	FRAN001	*F. tularensis *type A	A2	38 derivative (ATCC 6223)	Utah	1920 (?)	USAMRIID

25	FRAN027	*F. tularensis *type A	A2	38A (38 derivative)	Utah	-	USAMRIID

26	FRAN028	*F. tularensis *type A	A2	Larsen NIH38 (38 derivative)	Utah	-	USAMRIID

27	LVS	*F. tularensis *type B			Russia	1958 (?)	CDC

28	KY99-3387	*F. tularensis *type B			Kentucky	1999	CDC

29	OR96-0246	*F. tularensis *type B			Oregon	1996	CDC

30	OR96-0463	*F. tularensis *type B			Oregon	1996	CDC

31	KY00-1708	*F. tularensis *type B			Kentucky	2000	CDC

32	MO01-1673	*F. tularensis *type B			Missouri	2001	CDC

33	IN00-2758	*F. tularensis *type B			Indiana	2000	CDC

34	CA99-3992	*F. tularensis *type B			California	1999	CDC

35	FRAN004	*F. tularensis *type B		LVS	Russia	1958 (?)	USAMRIID

36	FRAN012	*F. tularensis *type B			Alabama	1991	USAMRIID

37	FRAN024	*F. tularensis *type B		JAP	Japan	1926	USAMRIID

38	FRAN025	*F. tularensis *type B		VT68	Vermont	1968	USAMRIID

39	FRAN029	*F. tularensis *type B		425	Montana	1941 (?)	USAMRIID

40	FRAN003	*F. novicida*		ATCC 15482 (U112)	Utah	1950	USAMRIID

^a^Strains characterized to the level of A1a or A1b by *Pme*I PFGE are indicated.

^b^Isolate recovered from a clinically normal rabbit

**Table 2:** *F. tularensis *strains used to evaluate SNP diagnostic markers

**S. No**.	Isolate	Subspecies	Clade	Geographic Source	Year isolated
1	ND00-0952	type A	A1 (A1a)	North Dakota	2000

2	MO01-1907	type A	A1 (A1a)	Missouri	2001

3	AR00-0028	type A	A1 (A1a)	Arkansas	2000

4	KS00-0948	type A	A1 (A1a)	Kansas	2000

5	OK01-2528	type A	A1 (A1a)	Oklahoma	2001

6	CA00-0036	type A	A1 (A1a)	California	2000

7	AR98-2146	type A	A1 (A1a)	Arkansas	1998

8	GA02-5497	type A	A1 (A1a)	Virginia	1982

9	NC01-5379	type A	A1 (A1b)	North Carolina	2001

10	NY04-2787	type A	A1 (A1b)	New York	2004

11	AK96-2888	type A	A1 (A1b)	Alaska	1996

12	OK02-0195	type A	A1 (A1b)	Oklahoma	2002

13	PA04-2790	type A	A1 (A1b)	Pennsylvania	2004

14	CA04-2258	type A	A1 (A1b)	California	2004

15	GA02-5375	type A	A1 (A1b)	New York	1977

16	WY03-1228	type A	A2	Wyoming	2003

17	CO01-3713	type A	A2	Colorado	2001

18	UT07-4362	type A	A2	Utah	2007

19	TX00-1591	type A	A2	Texas	2000

20	GA02-5453	type A	A2	Wyoming	1993

21	WY01-3911	type A	A2	Wyoming	2001

22	NM99-0295	type A	A2	New Mexico	1999

23	ID04-2687	type A	A2	Oregon	2004

24	AZ00-1180	type B		Arizona	2000

25	AZ00-1324	type B		Arizona	2000

26	SP03-1782	type B		Spain	2003

27	WA98-1774	type B		Washington	1998

28	E3443	type B		Oregon	1978

29	SP98-2108	type B		Spain	1998

30	OR98-0719	type B		Oregon	1998

31	RC503	type B		Russia	-

32	SP03-1783	type B		Spain	2003

33	CN98-5979	type B		Canada	1998

34	NY98-2295	type B		New York	1998

35	TX03-3834	type B		Mississippi	2003

36	IN00-2758	type B		Indiana	2000

37	F4853	type B		California	1983

38	OH01-3029	type B		Kansas	2001

39	CO05-3922	type B		Colorado	2005

### *Francisella *genomic DNA

Genomic DNAs of *F. tularensis *reference strains LVS and SCHU S4 were obtained from Dr. Luther Lindler of Global Emerging Infections Surveillance and Response System of Department of Defense. Genomic DNA was isolated from the strains in Table 1 and Table 2 using the QIAamp DNA mini kit or Gentra Puregene Cell Kit (Qiagen, Valencia, CA) according to the manufacturer's instructions. Genomic DNA samples were stored at -80°C.

### *F. tularensis *custom resequencing array set

The basis of the Affymetrix GeneChip^® ^resequencing by hybridization and the details of the design of *F. tularensis *GeneChip^® ^set has been described earlier [13]. Briefly, the design is primarily on the basis of the DNA sequence of strain LVS (GenBank Accession: AM 233362) serving as a reference and complemented with unique sequences of SCHU S4 (GenBank Accession: AJ 749949). A total of 1,764,558 queryable bases were identified for resequencing by hybridization after exclusion of ~9.22% of repetitive sequence from the design. This sequence was tiled onto a set of six CustomSeq 300 K GeneChips^® ^by Affymetrix, Inc., (Santa Clara, CA). This design provides approximately 91% of the *F. tularensis *double stranded genome sequence information from *holarctica *(type B) and *tularensis *(type A) subspecies. The whole genome resequencing was performed in duplicate for all query strains.

### Whole genome amplification, resequencing assay and raw data acquisition

*Francisella *genomic DNA amplification, DNA fragmentation, labeling, hybridization and acquisition of raw data was carried out exactly as described earlier [13].

### Processing of raw data with bioinformatic filters

Hybridization of a whole-genome sample on an Affymetrix^® ^resequencing array platform can lead to incorrect basecalls due to a number of systematic effects that are less prevalent when the sample consists of a purified PCR product. We have developed bioinformatic filters to account for most of these predictable adverse effects. Our bioinformatic filters consist of a set of Perl scripts that operate on the CHP files generated by GSEQ software and produce a list of high-confidence SNP calls from the larger raw set of SNPs calls present in those files. The scripts are available for download from our website http://pfgrc.jcvi.org/index.php/compare_genomics/snp_scripts.html. Each filter serves to reduce the number of candidate SNPs. The output of one filtering step becomes the input for the next. The detailed descriptions of these filters have been reported [13].

Briefly, the quality filter implemented in GSEQ software initially eliminates SNP calls that have been assigned low quality scores based on the difference in signal intensity between the highest intensity probe pair and the next highest intensity pair at a particular locus. The first filter applied is the "low homology filter" which identified regions that performed poorly as a result of deletions in the sample relative to the reference sequence. The base calls from the CHP files from GSEQ software are scanned to identify regions of adjacent positions that are rich in no-calls and SNP calls. SNP calls that occur within the defined low homology region are removed from the list of high-confidence SNP calls. The next script is referred to as the alternate homology filter. The alternate homology effect is caused by the sequences in the query DNA sample capable of hybridizing with high efficiency to more than one probe pair at a locus on the array. When a locus contains two strongly hybridizing probe pairs, the GSEQ software may make a SNP call, a reference base call or a no-call ("N"), depending on the relative signal strengths of the probe pairs. The alternate homology filter identifies SNP calls that may have arisen as a result of this effect based on the difference in binding energy between the alternate (SNP) sequence and the reference sequence. If the difference between these two binding energies is = 11.5 kcal/mol, the SNP call is assumed to be an artifact of the alternate sequence homology, and it is removed from the list of high confidence SNP calls. The remaining SNP calls are then put through the footprint effect filter. The artifact called the footprint effect is caused by the occurrence of a real SNP in a query sample that results in a destabilizing effect on 25-mers in the immediate vicinity of the SNP. The footprint effect filter algorithm assumes that a genuine SNP is most likely to cause spurious SNP calls at locations within 10 bases on either side of the genuine SNP. Any SNP call that occurs more than 10 base positions from the nearest neighboring SNP call is assumed to be valid, and any SNP call that has one or more neighbors within 10 base positions is subjected to the filter. Since any number of consecutive SNP calls within 10 base positions of each other may occur in the data, this filter is implemented as a recursive algorithm. For each list of consecutive SNP calls that each lies within 10 bases of its neighbors, the algorithm identifies the SNP call having the highest quality score. That SNP call is accepted as valid, and its immediate neighbors are removed from the list of high confidence SNP calls. This action may break the original list of neighboring SNP calls into two separate lists. All resulting lists are processed recursively in the same way, until all of the SNP calls have been accepted or rejected. This algorithm is implemented in the RemoveFootprintEffect.pl Perl program. All the above filters are applied to individual data sets generated for any sample, following which a final filter referred to as the replicate combination filter is applied. The replicate combination filter generates the list of common SNPs present in both the experiments.

### Phylogenetic clustering, selection of SNP markers and PCR primer design from multistrain global *Francisella *SNP collection

We generated a phylogenetic tree from the resequencing data by considering only those locations at which a SNP occurred in one or more of the forty strains. For each strain, we constructed a sequence containing the base calls at each of the locations at which a SNP was found in some strain(s). This resulted in forty sequences, each containing 19,897 base calls (including no-calls) which were used for the phylogenetic analysis. The phylogenetic tree was generated using the MrBayes program, version 3.1.2 [15-17]. The program was run for 200,000 generations, using a haploid model. The root of the resulting tree was inferred by midpoint rooting. The resulting tree is reported as a cladogram and as a phylogram. A phylogenetic tree (Additional File 1) was also generated from the same data using the dnaml (maximum likelihood) program of the PHYLIP package version 3.6 [18].

Node pairings which discriminated between subspecies or clades were selected for the development of diagnostic typing assays. Criteria used to select SNP locations for the assay were:

1. The SNP location must cleanly differentiate the two nodes of interest. Within each of the nodes, all of the member strains must share the same base call at the location, and the two nodes must differ at the location.

2. The sequences downstream of the SNP location must be in sufficient agreement among all strains from both nodes so that an appropriate primer can be chosen from the consensus sequence (the consensus at the primer location may not contain "N" calls or any conflicting base calls).

3. The primer sequences must have melting temperatures within a specific limited range (60°C to 70°C).

4. The predicted PCR product size must be within the range 150 to 500 bp.

We developed a set of programs to identify candidate SNP locations for the real-time PCR (RT-PCR) assay. SNPTree uses the phylogenetic tree and the multi-FASTA files from the resequencing experiments as input, assigns arbitrary node numbers to all nodes in the tree, and produces a set of multi-FASTA files, one for each node in the tree, of the consensus base calls for each node. The consensus call is "N" unless all members of a particular node share the same base call at that location. The program also produces a set of files, one for each node, listing the base calls that occur at every SNP location, for all SNP positions detected within the entire set of 40 samples (19,897 locations). The program CompareNodes uses the SNP list files for any two nodes and produces a list of SNP locations that cleanly differentiate the two nodes (described above). The program CreatePrimer3 uses a list of discriminating SNP locations and the multi-FASTA files for two nodes, and creates an input file for the Primer3 program [19]. CreatePrimer3 also chooses the 5'-forward primers, which are constrained by the locations of the SNPs. The Primer3 software [19] is then used to identify appropriate 3'-reverse primers. The Primer3 program enforces the last three criteria listed above. This process resulted in the design of a large number of primers for candidate SNP locations for most node pairs that may be used as diagnostic markers. The final set of SNP markers/locations we used was selected manually by identifying primers distributed over the entire genome. The programs SNPTree, CompareNodes and CreatePrimer3 were developed at the J. Craig Venter Institute specifically for this study and are freely available for download ftp://ftp.jcvi.org/pub/software/pfgrc/SNPTree/SNPTreePackage.tar.gz. These programs along with our bioinformatic filter pipeline can easily be adapted for other bacterial model systems for whole genome resequencing and SNP phylogeny using the Affymetrix resequencing array platform.

Primer3 software was used to design discriminating PCR primers based on the set of discriminating locations identified. Three primers were designed at each discriminating location: a 5'-forward primer with the node X call in the 3' position; a 5'-forward primer with the node Y call in the 3' position; and a single 3'-reverse primer. A base call at the discriminating location is determined by two PCR reactions where one of the two yields a lower cycle threshold (Ct) value. The RT-PCR primers used are shown in Additional File 2.

### Real-time PCR assays for *F. tularensis *typing

Real-time PCR assays to identify *F. tularensis *subspecies and clades were developed using SYBR^® ^Green (BioRad, Hercules CA) which binds all dsDNA molecules, emitting a fluorescent signal of a defined wavelength (522 nm). Reactions were performed in 20 μl volume and contained 80 pg of genomic DNA (0.01 ng/μl), 150 nM of forward and reverse primers and 10 μl of iQ SYBR^® ^Green Supermix (BioRad, Hercules CA). Reaction components were mixed in a V-bottom thin wall PCR 96-well plate (BioRad, Hercules CA). Real-time PCR was performed using the iCycler iQ (BioRad, Hercules, CA) with the following thermal cycling parameters: 50°C for 2 min, 95°C for 5 min, 60 cycles of 95°C for 15 seconds and 68°C for 30 seconds, 72°C for 30 seconds, 95°C for 1 min and finally 55°C for 3 min. The fluorescence was measured at 72°C in the cycle program. A cycle threshold (Ct) was automatically generated by the iCycler iQ Version 3.0a analysis software for each amplification reaction (BioRad, Hercules CA). Melt curve analysis was performed to verify that no primer dimers formed.

## Results

### Whole genome resequencing of strains

Previously, we reported an Affymetrix Inc. GeneChip^® ^array based whole genome resequencing platform for *F. tularensis*. Our whole-genome sequencing by hybridization approach made use of a set of bioinformatic filters to eliminate a majority of false positives and indicated a base call accuracy of 99.999% (Phred equivalent score 50) for type B strain LVS [13]. The base call accuracy was determined by comparing the base calls remaining after the application of our filters to the published sequence of the LVS strain. The bioinformatic filter programs may be accessed at http://pfgrc.jcvi.org/index.php/compare_genomics/snp_scripts.html. Two type A strains, WY96 3418 and SCHU S4 showed base call accuracies of 99.995% and 99.992% with Phred equivalent scores of 43 and 41 respectively [13]. We used this approach to collect whole-genome sequence and global SNP information from 40 *Francisella *strains. Table 1 shows the list of strains analyzed in this study. Twenty six type A (20 A1 and 6 A2), thirteen type B and one *F. novicida *strain were resequenced.

**Figure 1:**
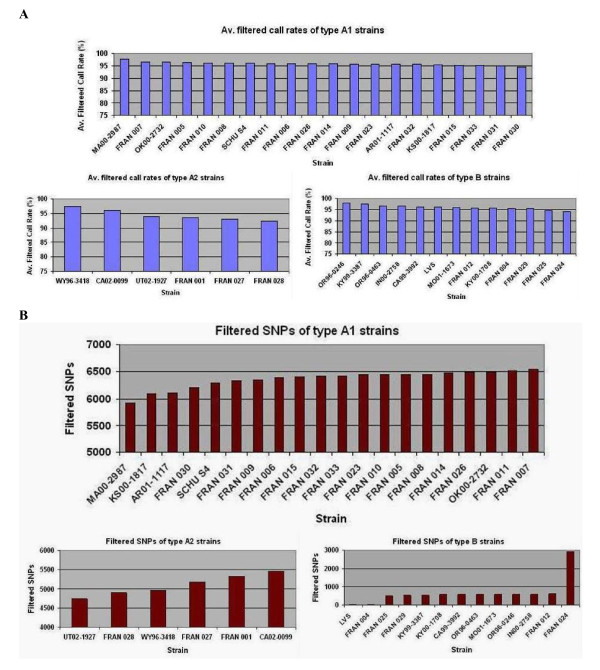
**Whole genome resequencing and SNP profiles of *F. tularensis *strains**. **(A) **Whole genome resequencing call rates and **(B) **single nucleotide polymorphic profiles of 39 *F. tularensis *type A and B strains. The data is an average of sample analysis performed in duplicate. The filtered base call rate and the filtered SNP values were obtained by processing the raw data from Affymetrix software through our bioinformatic filters [13]. Strains are displayed as either A1, A2 or type B for comparative analysis. *F. tularensis *subsp. *novicida *(FRAN003) displayed an average filtered base call rate of 83.041% and 12407 filtered SNPs (data not shown).

The base call rate and number of SNPs for *F. tularensis *A1, A2 and type B strains are shown in Figure 1 and Additional File 3. The base call rate for all forty strains was in the range of 83.04% to 97.92%. This range improved to 92.43% - 97.92% when the *F*. *novicida *strain FRAN003 (base call rate of 83.041% and total SNPs 12407) was excluded. The whole genome resequencing call rate was in the range of 94.62% to 97.62% for A1 strains, 92.43% to 97.41% for A2 strains and 94.04% to 97.92% for type B strains. Overall, type B strains displayed the highest average base call rate of 95.97% ± 1.06% and A2 displayed the lowest with 94.40% ± 0.64%. The average base call rate for A1 strains was 95.87% ± 0.64%. The total number of SNPs for all forty strains ranged widely from 15 to 12,407. As expected FRAN003, the *F*. *novicida *strain, displayed the highest number of SNPs (12,407) compared to the *F. tularensis *reference (LVS + SCHU S4) sequence. The wide range in SNP differences was reduced almost by half, 15 to 6543, when the *F. novicida *sequence was excluded.

*F. tularensis *type B strains displayed the lowest number of SNPs, ranging from 15 to 2915. As expected, LVS strains (LVS and FRAN004) showed the fewest SNP positions (15-16) when compared to the reference sequence. The genomes of all other type B strains, except for FRAN024, contained 497 - 605 SNPs, when compared to the reference sequence. FRAN024 showed a significantly higher number of SNPs (2915) compared to other type B strains. FRAN024 is a Japanese *holarctica *strain. It has been reported that the *F. tularensis *subsp. *holarctica *isolates from Japan are unique, being somewhat intermediate to *F. tularensis *subsp. *tularensis *and the other *F. tularensis *subsp. *holarctica *isolates [20,21]. A1 strains showed the highest number of SNPs when compared to the reference sequence with a range of 5929 to 6543 whereas A2 strains displayed a range of 4732 to 5469 SNPs. The average number of SNPs for A1 strains was 6362 ± 161 and 5096 ± 281 for A2 strains.

### Whole genome phylogenetic clustering of strains and SNP analysis

**Figure 2:**
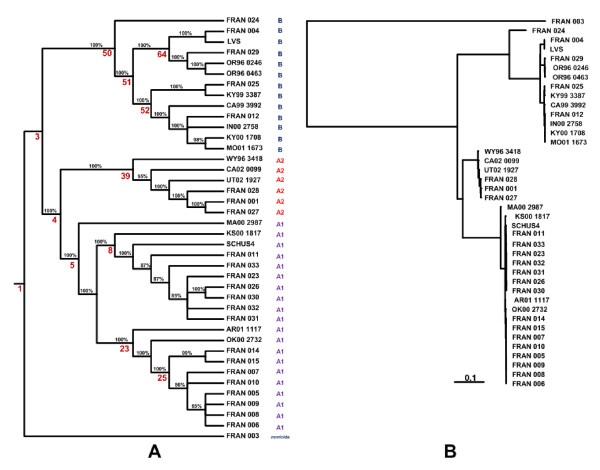
**Whole genome SNP based phylogenetic analysis of *Francisella *strains**. Phylogenetic analysis of resequenced *Francisella *strains. The whole-genome resequencing data was pared down to those base positions at which a SNP call occurred in one or more of the forty strains. These sequences were used to generate a phylogenetic tree using the MrBayes program as described in methods. This tree was then displayed as a cladogram (A) and as a phylogram (B) using the TreeView program http://taxonomy.zoology.gla.ac.uk/rod/treeview.html. Distinct clustering of type A and type B strains was observed. Both type A and B strains were further discriminated within the clusters. In the cladogram, the percentage values on the branches are the probabilities of the partitions indicated by each branch. The numbers shown in red are node numbers of significant nodes that are referenced in the manuscript. In the phylogram, the branch lengths are proportional to the mean of the posterior probability density, and a scale bar is given to relate the branch lengths to their numeric values.

**Figure 3:**
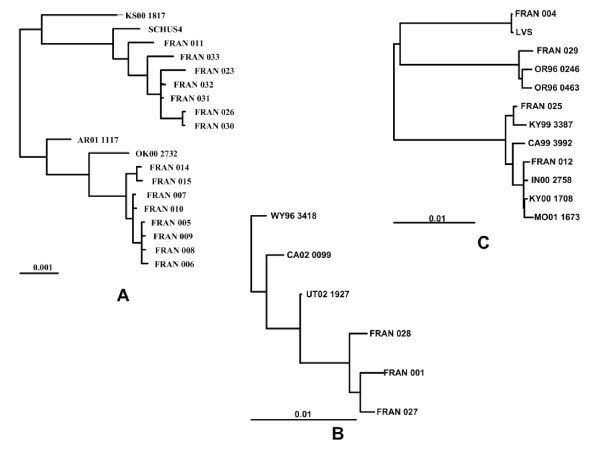
**Expanded phylogram for *F. tularensis *A1, A2 and type B strains**. Expanded sections of the phylogram (Figure 2B) containing the *F. tularensis *A1 strains except MA00 2987 (A), A2 strains (B) and type B strains except FRAN024 (C). The three subtrees are shown at different scales. The scale bars below each subtree are given to relate the branch lengths to their numeric probability values.

The cladogram and phylogram generated from the whole-genome resequence SNP data of all 40 *Francisella *strains is shown in Figure 2. Phylogenetic analysis revealed distinct clustering of the strains into the two subspecies, type A and type B, with further separation of strains within clusters. *F. novicida *(FRAN003) was distinct from type A and type B and formed its own phylogenetic group. Nodes (including internal nodes and leaf nodes) of the phylogenetic tree were assigned numbers by the SNPTree program. All type A strains emerged from node 4, whereas all type B strains emerged from node 50. The type A strains were divided into two primary sub-nodes, node 39 and node 5, corresponding to clades A2 and A1 respectively. A1 strains further subdivided into node 8, node 23, and node 5, corresponding to clades A1a and A1b and the MA00-2987 strain, respectively (Table 1). SCHU S4, the laboratory type A strain, fell within the A1a clade (node 8). Type B strains also divided into two clades based on nodes 52 and 64; these clades are referred to here as B1 and B2, respectively. The Japanese *holarctica *isolate FRAN024 formed its own phylogenetic group. Subsections of the phylogenetic tree at higher resolution, representing the type A1 (excluding MA00-2987), A2 and B strains (excluding FRAN024) are shown in Figure 3.

Within type A nodes, strains originating from distinct geographic locations (WY96 3418, CA02 0099, UT02 1927, KS00 1817, MA00 2987, AR01 1117, OK00 2732) with no known link to one another were clearly resolved by whole genome SNP based phylogenetic clustering (Figure 3, Table 1). This method also showed high potential for differentiating between closely related *F. tularensis *strains. The A1a strains, SCHU S4, FRAN023, FRAN031, FRAN032, FRAN026, FRAN030, and FRAN033 all originate from the same temporal location (Ohio) in the 1940's (Figure 3, Table 1). FRAN031 and FRAN032 could not be distinguished on the basis of SNPs, suggesting they may represent the same strain. Similarly, the A1b strains, FRAN005, FRAN006, FRAN007, FRAN008, FRAN009, FRAN010, FRAN014, and FRAN015 all derive from cottontail rabbit from one state park in Illinois, with 5 or fewer SNP differences distinguishing these strains (Figure 3, Table 1). The A2 strains, FRAN001, FRAN027 and FRAN028, were considered likely derivatives of the avirulent strain 38 (Jellison); SNP based phylogenetic clustering confirms this assumption (Figure 3, Table 1).

Within type B nodes, strains from Russia and North America were associated with node 64 (B2 strains), whereas only strains derived from North America (B1 strains) were associated with node 52 (Figure 3, Table 1). Overall, all unique type B strains (FRAN029, OR96 0246, OR96 0463, FRAN025, KY99 3387, CA99 3992, FRAN012, IN00 2758, KY00 1708 and MO01 1673) were resolved using whole genome SNP analysis.

Table 3 summarizes the SNP content for each of the major nodes identified in our phylogenetic analysis (Figure 2). The differentiating SNPs and maximum SNP separation numbers are indicators of the diversity within each node, as these represent SNP differences between members of the node (rather than SNP differences relative to the reference genome). The differentiating SNPs are the number of locations at which two or more member strains have differing base calls. Maximum SNP separation is the maximum number of SNP differences that are found between any two members of the node. As expected, the SNP diversity is greatest within subspecies (type A and type B) and decreases within clades; B1, A1a and A1b strains showed the least diversity (maximum SNP separation of 76, 75 and 38, respectively). Typing methods have previously revealed less diversity within type B than type A strains [2,21-23]. Similarly, our data show less diversity among type B isolates, with a maximum SNP separation of 602 when the Japanese *holarctica *strain FRAN024 is excluded from this analysis (B*). However, when all type B isolates, including the Japanese holarctica strain FRAN024, are included in the analysis, our data indicates a similar level of diversity for types A and B (maximum SNP separation of 2779 and 2833, respectively).

**Table 3:** SNP content of the major nodes identified in the phylogenetic tree (cladogram)

Node	Sub-species/clade/sub-clade	Number of strains per node	Total SNPs	Total SNPs in LVS genome	Total SNPs in SchuS4 unique sequence	Common SNPs	Unique SNPs	Differentiating SNPs	Maximum SNP separation
50	B	13	3771	3686	85	5	2837	3656	2833

51	B*	12	1154	1115	39	6	233	1060	602

52	B1	7	779	750	29	385	164	161	76

64	B2	5	705	677	28	7	153	628	549

4	A	26	8653	8559	94	2905	514	3765	2779

39	A2	6	6003	5919	84	3789	358	316	201

5	A1	20	7306	7291	15	4953	323	497	176

8	A1a	9	7001	6993	8	5491	277	129	75

23	A1b	10	7030	7022	8	5537	234	71	38

* contains all the type B strains with the exception of FRAN024, Japanese *holarctica *strain.

Total SNPs are locations at which a SNP occurs in one or more strains in the node (if the same SNP occurs in more than one strain, that location is counted only once). Common SNPs are locations where all strains in the node share the same base call, which is different from the reference call on the resequencing platform. Unique SNPs are locations where just a single strain in the node has a base call that differs from the reference sequence. Differentiating SNPs are locations at which at least two strains in the node have different base calls. Maximum SNP separation is the number of base calls separating the two most distant members of the node. Differentiating SNPs and maximum SNP separation are both indicators of the degree of diversity within the node. The detection of diversity is limited by the extent to which our sample set is representative of the variability within each clade in nature. Refer to Figure 2 for the details of strain clustering.

The presence of a large number of differentiating SNPs within each phylogenetic node suggests that a deeper level of discrimination can be achieved by identifying SNPs unique to individual strains. The smallest number of differentiating SNPs within a phylogenetic node was 71 (A1b strains). The phylogram (Figure 2B) indicates that the closest clade pairings are between A1a/A1b and B1/B2 which is quantitatively in agreement with the SNP differences as shown in Additional File 4. Phylogenetic analyses performed by two independent approaches (Bayesian in Figure 2 and maximum likelihood in Additional File 1) showed some differences only at the level of minor clades in the trees. These did not affect the subsequent analyses.

### Typing assays based on high quality global SNP markers

**Figure 4:**
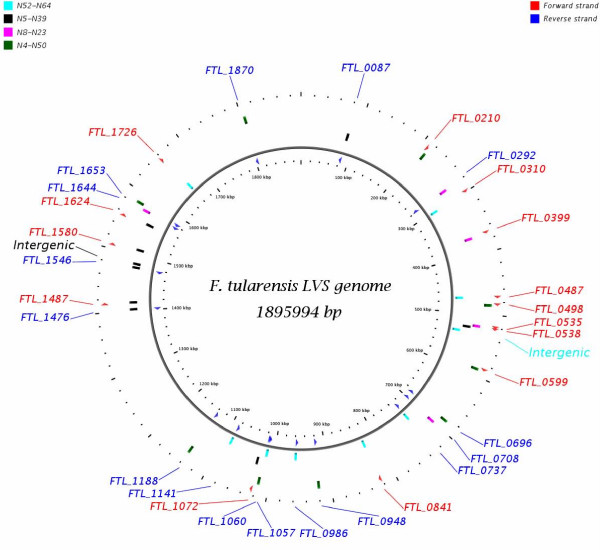
**Location of *in silico *identified diagnostic SNP markers in the *F. tularensis *LVS genome**. Representation of *in silico *discriminating SNP markers on the *F. tularensis *LVS genome. The vertical colored bar represents the position of the SNP marker on the LVS with the relevant node pair indicated by color. Loci containing the discriminatory SNP markers in the forward and reverse strands are shown in red and blue respectively. Two markers in the non-coding sequences of the genome are also shown.

Node pairings that discriminated between *F. tularensis *subspecies or within subspecies were selected for the development of SNP diagnostic typing assays (Figure 2). The four node pairings were node 4 and node 50, node 52 and node 64, node 39 and node 5, and node 8 and node 23 for discrimination of type A vs. type B, B1 vs. B2, A2 vs. A1 and A1a vs. A1b, respectively. A SNP location was selected to differentiate between two nodes in the tree when all strains belonging to one node contain the SNP call and all strains belonging to the other node contain the reference call at that location. The location of the 32 *in silico *identified diagnostic SNP markers in the *F. tularensis *LVS genome are shown in Figure 4. Fourteen SNP loci were in the forward strand, sixteen in the reverse and two loci were in non-coding intergenic regions. The discriminating nodes, SNP location, locus name, gene symbol with product and the role category is described in the Additional File 5.

To show that SNPs can be used as diagnostic markers for typing of *F. tularensis *subspecies and clades, RT-PCR assays were designed. Initially, seven *F. tularensis *strains were used to screenthe 32 RT-PCR discriminatory SNP positions for the ability to distinguish type A vs. type B, A1 vs. A2, A1a vs. A1b, and B1 vs. B2. Preliminary results indicated 5 out of 9 primer sets (684048, 917759, **1014623, 1136971**, 1581977) distinguished type A and type B, 3 out of 9 primer sets distinguished A1 and A2 (**521982**, 1025460, **1507435**), 2 out of 5 primers sets distinguished A1a and A1b (**518892, 1574929**) and 3 out of 9 primer sets distinguished B1 and B2 (**299153, 470635**, 1011425). The two primer sets from each group displaying the largest difference in Ct values (shown in bold) were pursued further (1014623, 1136971, 521982, 1507435, 518892, 1574929, 299153 and 470635). To determine the robustness of these discriminatory SNP positions, an additional 39 *F. tularensis *strains (23 type A, 16 type B) (Table 2) were examined.

**Figure 5:**
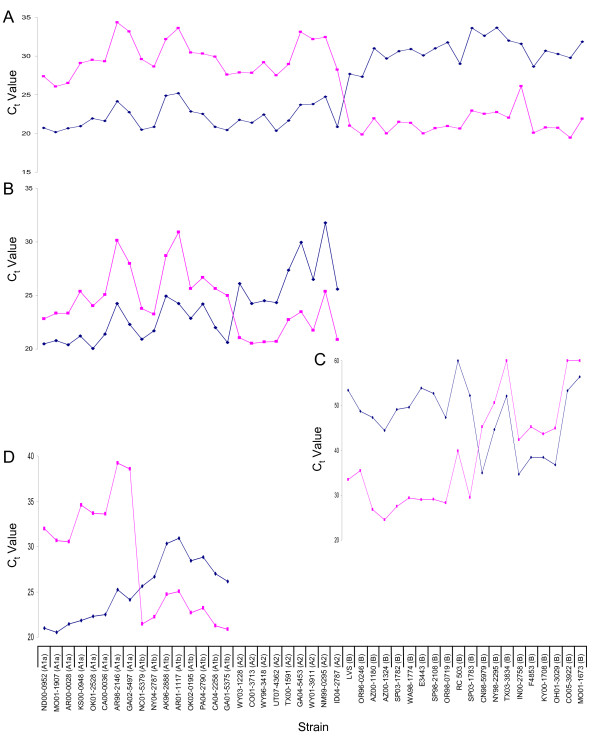
**Real-time PCR evaluation of SNP diagnostic markers**. Evaluation of SNP diagnostic markers using real-time PCR. Data is shown for primer sets **A) **1014623 discriminating node pairings 4 and 50 (type A vs. type B); **B) **521982 discriminating node pairings 5 and 39 (A1 vs. A2); **C) **299153 discriminating node pairings 52 and 64 (B1 vs. B2); and **D) **1574929 discriminating node pairings 8 and 23 (A1a vs. A1b). The six control strains included in the analysis are also shown; A1 (AR01 1117), A2 (WY96 3418), B1 (LVS, OR96 0246) and B2 (KY00 1708, MO01 1673).

The data for 4 primer sets (1014623, 521982, 299153 and 1574929) is shown in Figure 5. These assays are hierarchical in nature. The first primer set determines whether a strain is type A or type B based on SNP 1014623. In type A and type B strains, this nucleotide position is T and C, respectively. A strain identified as type B can be further typed as B1 or B2 based on SNP 299153 (G in B1 strains and T in B2 strains). Similarly, strains identified as type A can be classified as A1 or A2 based on SNP 521982 (T in A1 strains and C in A2 strains) and A1 strains further characterized as A1a or A1b by SNP 1574929 (G in A1a strains and C in A1b strains).

As shown in Figure 5, the type A and type B SNP assay clearly distinguished between the 23 type A and 16 type B strains. The 23 type A strains were then subdivided into 15 A1 and 8 A2 strains and the 15 A1 strains were subsequently further sub-divided into 8 A1a and 7 A1b strains. For all 23 type A strains, the classification of strains as A1, A2, A1a or A1b by diagnostic SNP typing corresponds with *Pme*I PFGE typing results (Table 2) [14], emphasizing the power and the utility of this simpler methodology for classification of type A clades.

Type B strains were also resolved into B1 and B2 clades based on a single SNP. As these clades were newly identified by our SNP based phylogenetic clustering, resequenced B1 (KY00 1708 and MO01-1673) and B2 (LVS, OR96 0246) strains were included as positive controls. Of the 16 type B strains tested, nine isolates were classified as B2 and 7 isolates were classified as B1. Isolates from Russia (RC 503), Spain (SP03 1782 and SP98 2108) Finland (SP03 1783) and the US were identified as B2 by this assay, whereas isolates from Canada and the US were identified as B1, providing evidence for geographic clustering of type B isolates based on this SNP marker. In summary, this work shows the potential for development of SNP typing markers based on a relatively small number of "complete" genome sequences. For future work, it will be important to define a set of SNPs that could be used for high-resolution discrimination to the strain level.

## Discussion

Whole genome comparative analysis and collection of high-confidence global SNPs from multiple strains of a given bacterial species has a number of applications in both basic and translational research. Our study was undertaken with an objective of providing the scientific community with whole-genome sequence and SNP information from multiple strains of *F. tularensis*, enabling rapid advancements in our understanding of basic and applied biology of this organism. *F. tularensis *has been recognized as a causative agent of tularemia for almost a century [24] and is classified as a category A biodefense agent. We have collected nearly complete (~91%) genome sequence and global SNP information from forty *Francisella *strains using our whole genome high-density resequencing array platform [13]. All the sequence and SNP information is publicly available to the scientific community from Biodefense and Public Health Database (BioHealthBase) at http://www.biohealthbase.org/GSearch/home.do?decorator=Francisella. BioHealthBase is a Bioinformatics Resource Center (BRC) for biodefense and emerging/re-emerging infectious diseases that is supported by the National Institute of Allergy and Infectious Diseases (NIAID). The data can also be obtained from our web site at http://pfgrc.jcvi.org/index.php/compare_genomics/francisella_genotyping.html or through the JCVI ftp server at ftp://ftp.jcvi.org/pub/data/PFGRC/Ft_DataRelease/. This multi-strain high-quality nearly complete genome sequence and global SNP information provides a unique opportunity to perform comparative genome analysis between *F. tularensis *strains, thus contributing towards a better understanding of pathogenicity and evolutionary relationships of this species. We have used this information to build a robust whole genome based phylogeny that enabled the identification of SNP discriminatory markers. We further validated high quality global SNP markers for typing of *F. tularensis *subspecies and clades as a proof of concept that these markers may be used for future development of high-resolution typing methods.

Previous reports have suggested that greater genetic diversity exists among type A as compared to type B strains [2]. Our whole genome SNP based analysis of 12 type B isolates from North America and Russia appears to confirm this observation. However, SNP data obtained after inclusion of a Japanese type B strain (FRAN024) indicated a similar level of SNP diversity in type A and type B strains (Table 3). Sufficient SNP diversity was observed among type B strains to generate an internal structure in the phylogenetic tree (Figure 2) as well as to resolve all unique strains. The single *F. novicida *isolate in our study, FRAN003 (U112), had the lowest base call rate (83.041%) and the highest number of SNPs (12,407) among our samples. The low base call rate is a likely reflection of the sequence divergence between the *F. novicida *strain (U112) and the reference sequence on our resequencing chips. Rohmer et. al[11]. have reported a nucleotide sequence identity of 97.8% between the LVS and *F. novicida *U112 genomes. The differences in these two approaches may be due to the fact that array-based resequencing is sensitive to sequence divergence, and performs best with samples that are homologous with the reference sequence. In our global SNP phylogenetic analysis, *F. novicida *(U112) is well separated from the *F. tularensis *isolates (Figure 2B).

A number of molecular approaches have been used to better understand the diversity of *Francisella *[2,21,25-27]. New subdivisions within *F. tularensis *subspecies have been revealed by these approaches. Differing methods provide differing resolution as most of the methods sample only a subset of the whole genome in order to assess relationships among different isolates [2]. MLVA is considered to provide the highest discriminatory power (i.e. strain level) [2,21,28]. PFGE typing has been used to identify four distinct type A genotypes, A1a, A1b, A2a and A2b [9], not previously observed by MLVA typing. PFGE typing combined with epidemiologic data revealed that the observed genetic diversity among type A strains correlated with differences in clinical outcome and geographic distribution. A1b strains were associated with significantly higher mortality in humans as compared to A1a, A2 or type B strains. Type B strains display little or no genetic diversity by PFGE [14] and a number of other molecular methods [2,10,21-23].

Comparative whole-genome sequence analysis provides the highest level of discrimination among different strains, but has not been widely used due to the high cost of this method. Keim et al [2] have shown a whole-genome SNP phylogeny of *Francisella *using ~8000 syntenic SNPs from the published whole genome sequences of seven strains. Use of only two type A and two type B genomes was sufficient to reveal that type A strains differ greatly from each other unlike type B strains. More recently, the phylogenetic structure of *F. tularensis *has been reported based on whole genome SNP analysis of thirteen publicly available genome sequences; 29,774 SNPs were used in this analysis [4]. In this study, we have constructed a phylogenetic profile of forty *Francisella *strains based on whole genome sequences. This to our knowledge is the first report of a phylogenetic model based on nearly complete genomes of multiple strains of *F. tularensis *using Affymetrix resequencing arrays.

We have demonstrated that resequencing data may be used to generate high-resolution phylogenetic trees based on global SNPs. The advantage of this sequence-based approach is that SNP based phylogenetic trees can be used for evolutionary analyses. The comparative analysis based on the phylogenetic relatedness of strains can provide significant insights into the varying degree of phenotypes and ecotypes of an organism. The total number of complete genomes required to achieve an optimum phylogenetic profile from the multiple strains of an organism will be determined by the degree of plasticity of the genome. Adequate phylogenetic relationship can be determined with a sufficient number of genomes from diverse isolates of an organism and the whole genome comparative analysis of such related strains can provide real biological insights into the adaptation and evolution of a species. Such phylogenetic-based comparative analysis can capture genomic differences of very closely related strains and provide valuable information for the development of rapid molecular sequence based assays, capable of discrimination to the strain level.

## Conclusion

The whole genome resequencing array platform provides sequence and SNP information from multiple strains for any infectious agent with an available whole genome sequence. Multi-strain whole genome sequence data allows one to build robust phylogenetic models for an organism based on global SNPs. Whole genome SNP based phylogenetic trees can guide meaningful comparative analysis of strains to better understand the biology of an organism as well as in translational research such as in developing high resolution economical SNP based typing assays. We have collected whole genome sequence and SNP information from forty strains of *Francisella *to construct a global phylogeny. Our data shows a good correlation with the previously published reports using limited genomic sequence information and also provides higher strain resolution. We used the whole genome SNP phylogeny to identify informative SNP markers specific to major nodes in the tree and to develop a genotyping assay for subspecies and clades of *F. tularensis *strains. Less diverse type B strains could even be discriminated into two clades, B1 and B2, based on a single SNP. Our whole genome SNP based phylogenetic clustering shows high potential for identifying SNP markers within *F. tularensis *capable of discriminating to the strain level. This finding should greatly facilitate the rapid and low-cost typing of *F. tularensis *strains in the future.

## List of abbreviations

DNA: deoxyribonucleic acid; LVS: live vaccine strain; MLVA: multi-locus variable number tandem repeat analysis; PFGE: pulsed-field gel electrophoresis; SNP: single nucleotide polymorphism; subsp.: subspecies; RT PCR: real-time polymerase chain reaction.

## Authors' contributions

GAP- planned, developed and co-coordinated the project, analyzed the data, wrote the manuscript; MHH - bioinformatic tool development and data analysis, contributed to the progress of the project and manuscript writing; JMP - contributed to the data analysis and manuscript preparation; SP- wet lab analysis, performed resequencing assays of the samples; SAK- bioinformatic data analysis, preparation of tables and figures; MJW- contributed to the data analysis and manuscript preparation; CM- data collection for the SNP typing assay of samples; MJ- contribution towards development and optimization of the SNP typing assay; MES-participated in data analysis and manuscript preparation; RDF-project oversight; SNP-project design, manuscript contribution and project oversight. All authors read and approved the final manuscript.

## Supplementary Material

Additional file 1 Whole genome SNP based phylogenetic analysis of *Francisella *strains using maximum likelihood method Click here for file

Additional file 2 List of RT- PCR primers for diagnostic typing assays Click here for file

Additional file 3 Whole genome resequencing call rates and SNPs for *F. tularensis *strains Click here for file

Additional file 4 Quantitative SNP differences between the major phylogenetic nodes in the cladogram Click here for file

Additional file 5 Features of *in silico *identified SNP diagnostic markers. Click here for file
