# Supplementary material for: Whole genome single nucleotide polymorphism based phylogeny of Francisella tularensis and its application to the development of a strain typing assay
Source: BMC Microbiol. 2009 Oct 7;9:213. doi: 10.1186/1471-2180-9-213 (PMC2767358; doi:10.1186/1471-2180-9-213)
Supplement: Additional file 1 — Whole genome SNP based phylogenetic analysis of Francisella strains using maximum likelihood method [file 1471-2180-9-213-S1.DOC]

**Additional File 1** Whole genome SNP based phylogenetic analysis of *Francisella* strains using maximum likelihood method


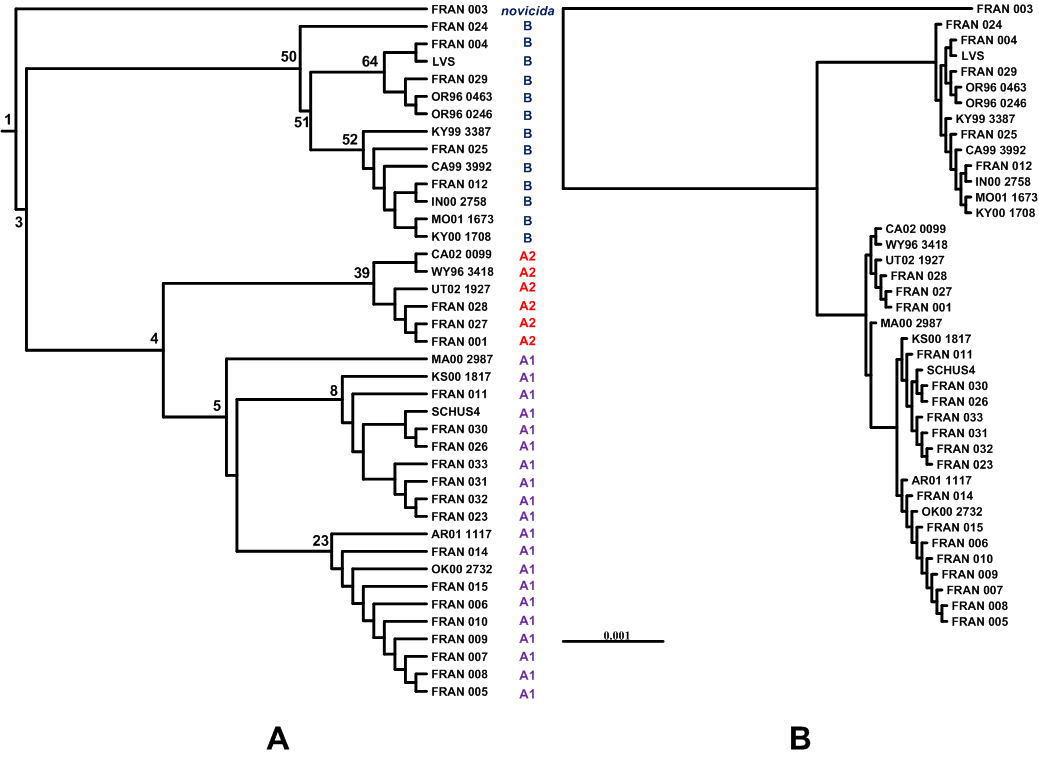


Phylogenetic analysis of resequenced *Francisella* strains. The whole genome resequencing data was compressed to represent base positions at which a SNP call occurred in one or more of the forty strains. The compressed sequences were used to generate a phylogenetic tree using the dnaml (maximum likelihood) program of the PHYLIP package as described in methods. This tree was then displayed as a cladogram (A) and as a phylogram (B) using the TreeView program (<http://taxonomy.zoology.gla.ac.uk/rod/treeview.html>). This method showed the distribution of all the forty strains into major clades similar to the data obtained using MrBayes program.
