# Supplementary material for: Whole genome single nucleotide polymorphism based phylogeny of Francisella tularensis and its application to the development of a strain typing assay
Source: BMC Microbiol. 2009 Oct 7;9:213. doi: 10.1186/1471-2180-9-213 (PMC2767358; doi:10.1186/1471-2180-9-213)
Supplement: Additional file 2 — List of RT- PCR primers for diagnostic typing assays [file 1471-2180-9-213-S2.DOC]

**Additional File 2:** List of RT-PCR primers for diagnostic typing assay

| **S. No.** | **SNP chip position** | **SNP genome position** | **Plate/well ID** | | | **Primer name** | **Primer sequence** | **Product size (~bp)** |
| --- | --- | --- | --- | --- | --- | --- | --- | --- |
| **1** | **1014623** | **1014621** | **SNP1AA** | **A** | **1** | **N4L_1014623** | **TGAACCACCTGTAGAAGCAGCATTT** | **477** |
| **2** | **1014623** | **1014621** | **SNP1AA** | **A** | **2** | **N50L_1014623** | **TGAACCACCTGTAGAAGCAGCATTC** | **477** |
| **3** | **1014623** | **1014621** | **SNP1AA** | **A** | **3** | **N4-50R_1014623** | **CGCCTAAGACACTGACAAAGAGTC** | **477** |
| **4** | **1136971** | **1136969** | **SNP1AA** | **A** | **4** | **N4L_1136971** | **GTGCTCCCATCTCAAACCAAAAATT** | **424** |
| **5** | **1136971** | **1136969** | **SNP1AA** | **A** | **5** | **N50L_1136971** | **GTGCTCCCATCTCAAACCAAAAATC** | **424** |
| **6** | **1136971** | **1136969** | **SNP1AA** | **A** | **6** | **N4-50R_1136971** | **CACCTGGATATTCTCTTGCTGTTG** | **424** |
| **7** | **1581977** | **1581975** | **SNP1AA** | **A** | **7** | **N4L_1581977** | **AAGCTGCTTGGCTCTATATCTACCT** | **225** |
| **8** | **1581977** | **1581975** | **SNP1AA** | **A** | **8** | **N50L_1581977** | **AAGCTGCTTGGCTCTATATCTACCC** | **225** |
| **9** | **1581977** | **1581975** | **SNP1AA** | **A** | **9** | **N4-50R_1581977** | **GGACATTATTGCGATAAAGAAGCTG** | **225** |
| **10** | **1802570** | **1802567** | **SNP1AA** | **A** | **10** | **N4L_1802570** | **TCTGCTTAAACTCATCATCTGTGGG** | **270** |
| **11** | **1802570** | **1802567** | **SNP1AA** | **A** | **11** | **N50L_1802570** | **TCTGCTTAAACTCATCATCTGTGGA** | **270** |
| **12** | **1802570** | **1802567** | **SNP1AA** | **A** | **12** | **N4-50R_1802570** | **AGCAAGATGGTGTTTTGCTTG** | **270** |
| **13** | **211514** | **211513** | **SNP1AA** | **B** | **1** | **N4L_211514** | **TGTTAAAGATGTTGCCGATAAGGAT** | **364** |
| **14** | **211514** | **211513** | **SNP1AA** | **B** | **2** | **N50L_211514** | **TGTTAAAGATGTTGCCGATAAGGAC** | **364** |
| **15** | **211514** | **211513** | **SNP1AA** | **B** | **3** | **N4-50R_211514** | **TCCCAAAGCCTCTTTCTTCTCAAG** | **364** |
| **16** | **482885** | **482885** | **SNP1AA** | **B** | **4** | **N4L_482885** | **CTCTTGATGGAGGATTATTTGTCCT** | **320** |
| **17** | **482885** | **482885** | **SNP1AA** | **B** | **5** | **N50L_482885** | **CTCTTGATGGAGGATTATTTGTCCC** | **320** |
| **18** | **482885** | **482885** | **SNP1AA** | **B** | **6** | **N4-50R_482885** | **TGCCACTAAAATAGTAAATGGCTTCTC** | **320** |
| **19** | **587086** | **587086** | **SNP1AA** | **B** | **7** | **N4L_587086** | **TTGGAAAAACACGAAAAACTTTATC** | **244** |
| **20** | **587086** | **587086** | **SNP1AA** | **B** | **8** | **N50L_587086** | **TTGGAAAAACACGAAAAACTTTATT** | **244** |
| **21** | **587086** | **587086** | **SNP1AA** | **B** | **9** | **N4-50R_587086** | **CCGCTATTAGATAACATTGGCTG** | **244** |
| **22** | **684048** | **684048** | **SNP1AA** | **B** | **10** | **N4L_684048** | **TTGCTGTGAGACAAACTCAACACTG** | **489** |
| **23** | **684048** | **684048** | **SNP1AA** | **B** | **11** | **N50L_684048** | **TTGCTGTGAGACAAACTCAACACTA** | **489** |
| **24** | **684048** | **684048** | **SNP1AA** | **B** | **12** | **N4-50R_684048** | **GCTGATAACCCAATCAAGCG** | **489** |
| **25** | **917759** | **917759** | **SNP1AA** | **C** | **1** | **N4L_917759** | **TGGTCTTCGAGCAGAGCTATTTTAC** | **232** |
| **26** | **917759** | **917759** | **SNP1AA** | **C** | **2** | **N50L_917759** | **TGGTCTTCGAGCAGAGCTATTTTAA** | **232** |
| **27** | **917759** | **917759** | **SNP1AA** | **C** | **3** | **N4-50R_917759** | **ACTTCAATGGTGAAGATGAAGAGTC** | **232** |
| **28** | **1025460** | **1025458** | **SNP1AA** | **C** | **4** | **N5L_1025460** | **GAGTGAATATGACACCTTTGGAGTT** | **396** |
| **29** | **1025460** | **1025458** | **SNP1AA** | **C** | **5** | **N39L_1025460** | **GAGTGAATATGACACCTTTGGAGTC** | **396** |
| **30** | **1025460** | **1025458** | **SNP1AA** | **C** | **6** | **N5-39R_1025460** | **TCAAAGAAATTTGCTGGTACAAACATC** | **396** |
| **31** | **1401061** | **1401057** | **SNP1AA** | **C** | **7** | **N5L_1401061** | **TTATGCTCATACAAAGCAATCAAGG** | **233** |
| **32** | **1401061** | **1401057** | **SNP1AA** | **C** | **8** | **N39L_1401061** | **TTATGCTCATACAAAGCAATCAAGA** | **233** |
| **33** | **1401061** | **1401057** | **SNP1AA** | **C** | **9** | **N5-39R_1401061** | **AACCATCAGCAGGCTTTACTTG** | **233** |
| **34** | **1413068** | **1413064** | **SNP1AA** | **C** | **10** | **N5L_1413068** | **AAATATTGCTAATAAACGTGTTGCC** | **431** |
| **35** | **1413068** | **1413064** | **SNP1AA** | **C** | **11** | **N39L_1413068** | **AAATATTGCTAATAAACGTGTTGCT** | **431** |
| **36** | **1413068** | **1413064** | **SNP1AA** | **C** | **12** | **N5-39R_1413068** | **GACTGAAGCTTGCGACAGCAG** | **431** |
| **37** | **1482239** | **1482237** | **SNP1AA** | **D** | **1** | **N5L_1482239** | **TCATGTGTTGGATACCTGAAAATTT** | **499** |
| **38** | **1482239** | **1482237** | **SNP1AA** | **D** | **2** | **N39L_1482239** | **TCATGTGTTGGATACCTGAAAATTG** | **499** |
| **39** | **1482239** | **1482237** | **SNP1AA** | **D** | **3** | **N5-39R_1482239** | **AAAAAGGCACAACAGGTAATCAATAG** | **499** |
| **40** | **1476018** | **1476016** | **SNP1AA** | **D** | **4** | **N5L_1476018** | **CAATGACTCTAAGATTTGTGCTTCA** | **255** |
| **41** | **1476018** | **1476016** | **SNP1AA** | **D** | **5** | **N39L_1476018** | **CAATGACTCTAAGATTTGTGCTTCG** | **255** |
| **42** | **1476018** | **1476016** | **SNP1AA** | **D** | **6** | **N5-39R_1476018** | **GGTTTAGCAGAGATGTTAAAGGGTGG** | **255** |
| **43** | **1507435** | **1507433** | **SNP1AA** | **D** | **7** | **N5L_1507435** | **ACGTCATACAACAACTTGCTCAACA** | **200** |
| **44** | **1507435** | **1507433** | **SNP1AA** | **D** | **8** | **N39L_1507435** | **ACGTCATACAACAACTTGCTCAACC** | **200** |
| **45** | **1507435** | **1507433** | **SNP1AA** | **D** | **9** | **N5-39R_1507435** | **TTTCACAACCCTTAGATCCTTCTTC** | **200** |
| **46** | **1555228** | **1555226** | **SNP1AA** | **D** | **10** | **N5L_1555228** | **CCATTTGCGAAATTTACCTTTTACC** | **297** |
| **47** | **1555228** | **1555226** | **SNP1AA** | **D** | **11** | **N39L_1555228** | **CCATTTGCGAAATTTACCTTTTACT** | **297** |
| **48** | **1555228** | **1555226** | **SNP1AA** | **D** | **12** | **N5-39R_1555228** | **CCATGCTTGAATTGCGTATGAC** | **297** |
| **49** | **521982** | **521982** | **SNP1AA** | **E** | **1** | **N5L_521982** | **CAAGCAACGGCAATACTTGGTGAAT** | **351** |
| **50** | **521982** | **521982** | **SNP1AA** | **E** | **2** | **N39L_521982** | **CAAGCAACGGCAATACTTGGTGAAC** | **351** |
| **51** | **521982** | **521982** | **SNP1AA** | **E** | **3** | **N5-39R_521982** | **TCCCAATAACACAGAATGGCCC** | **351** |
| **52** | **82272** | **82272** | **SNP1AA** | **E** | **4** | **N5L_82272** | **ATGCGCTACAGGTATAATTGGTATA** | **301** |
| **53** | **82272** | **82272** | **SNP1AA** | **E** | **5** | **N39L_82272** | **ATGCGCTACAGGTATAATTGGTATG** | **301** |
| **54** | **82272** | **82272** | **SNP1AA** | **E** | **6** | **N5-39R_82272** | **TTTTGTATTTTATGGTCTGGTACGC** | **301** |
| **55** | **1011425** | **1011423** | **SNP1AA** | **E** | **7** | **N52L_1011425** | **TGAAGTACACGCTTAATGTCATCAA** | **348** |
| **56** | **1011425** | **1011423** | **SNP1AA** | **E** | **8** | **N64L_1011425** | **TGAAGTACACGCTTAATGTCATCAG** | **348** |
| **57** | **1011425** | **1011423** | **SNP1AA** | **E** | **9** | **N52-64R_1011425** | **CCGCTTTTAGAATGGGTTTTTCAC** | **348** |
| **58** | **1083612** | **1083610** | **SNP1AA** | **E** | **10** | **N52L_1083612** | **TGTTGAACGGTCATTCCAATCTAGA** | **321** |
| **59** | **1083612** | **1083610** | **SNP1AA** | **E** | **11** | **N64L_1083612** | **TGTTGAACGGTCATTCCAATCTAGG** | **321** |
| **60** | **1083612** | **1083610** | **SNP1AA** | **E** | **12** | **N52-64R_1083612** | **GAAACGACAAGTTACATGGCTACAC** | **321** |
| **61** | **1659094** | **1659091** | **SNP1AA** | **F** | **1** | **N52L_1659094** | **TCCGCTGTTTAGAATTGATATTGGC** | **403** |
| **62** | **1659094** | **1659091** | **SNP1AA** | **F** | **2** | **N64L_1659094** | **TCCGCTGTTTAGAATTGATATTGGT** | **403** |
| **63** | **1659094** | **1659091** | **SNP1AA** | **F** | **3** | **N52-64R_1659094** | **TGATCCTGGTAAATGTTTTGAATGG** | **403** |
| **64** | **299153** | **299153** | **SNP1AA** | **F** | **4** | **N52L_299153** | **TGATCCTGTTGTTGATTTTGCTAAT** | **259** |
| **65** | **299153** | **299153** | **SNP1AA** | **F** | **5** | **N64L_299153** | **TGATCCTGTTGTTGATTTTGCTAAG** | **259** |
| **66** | **299153** | **299153** | **SNP1AA** | **F** | **6** | **N52-64R_299153** | **TCTTGGGAACTCTTGTAATGCAAC** | **259** |
| **67** | **530990** | **530990** | **SNP1AA** | **F** | **7** | **N52L_530990** | **TTATAAACTTGGCGACACTTGTTGG** | **222** |
| **68** | **530990** | **530990** | **SNP1AA** | **F** | **8** | **N64L_530990** | **TTATAAACTTGGCGACACTTGTTGA** | **222** |
| **69** | **530990** | **530990** | **SNP1AA** | **F** | **9** | **N52-64R_530990** | **TGGCTCAAATGGTTTTGGTTACG** | **222** |
| **70** | **470635** | **470635** | **SNP1AA** | **F** | **10** | **N52L_470635** | **AGATCAGACTGCAAAACTTCCAGAG** | **267** |
| **71** | **470635** | **470635** | **SNP1AA** | **F** | **11** | **N64L_470635** | **AGATCAGACTGCAAAACTTCCAGAA** | **267** |
| **72** | **470635** | **470635** | **SNP1AA** | **F** | **12** | **N52-64R_470635** | **CCTGTTCCAGGAATACCTAGTGATG** | **267** |
| **73** | **726518** | **726518** | **SNP1AA** | **G** | **1** | **N52L_726518** | **CAAAAATGTATTTTAAGGCAGCAAA** | **203** |
| **74** | **726518** | **726518** | **SNP1AA** | **G** | **2** | **N64L_726518** | **CAAAAATGTATTTTAAGGCAGCAAC** | **203** |
| **75** | **726518** | **726518** | **SNP1AA** | **G** | **3** | **N52-64R_726518** | **AATTTTCTCATTTATTATTTGCGTGAC** | **203** |
| **76** | **956165** | **956165** | **SNP1AA** | **G** | **4** | **N52L_956165** | **ACCAGCATGATCTATGAAAAACTGC** | **498** |
| **77** | **956165** | **956165** | **SNP1AA** | **G** | **5** | **N64L_956165** | **ACCAGCATGATCTATGAAAAACTGT** | **498** |
| **78** | **956165** | **956165** | **SNP1AA** | **G** | **6** | **N52-64R_956165** | **GCTGCATATATGGACATATCACACCC** | **498** |
| **79** | **823355** | **823355** | **SNP1AA** | **G** | **7** | **N52L_823355** | **AGAAAGGATTACCATCAGGAGCAAC** | **221** |
| **80** | **823355** | **823355** | **SNP1AA** | **G** | **8** | **N64L_823355** | **AGAAAGGATTACCATCAGGAGCAAT** | **221** |
| **81** | **823355** | **823355** | **SNP1AA** | **G** | **9** | **N52-64R_823355** | **TTTTGTATCCCATGTAGCAAGCG** | **221** |
| **82** | **1574929** | **1574927** | **SNP1AA** | **G** | **10** | **N8L_1574929** | **TGGCTTATCGCCGACATTCATCAAG** | **332** |
| **83** | **1574929** | **1574927** | **SNP1AA** | **G** | **11** | **N23L_1574929** | **TGGCTTATCGCCGACATTCATCAAC** | **332** |
| **84** | **1574929** | **1574927** | **SNP1AA** | **G** | **12** | **N8-23R_1574929** | **TGATTTGGAATCTAACTCGCGG** | **332** |
| **85** | **277706** | **277706** | **SNP1AA** | **H** | **1** | **N8L_277706** | **CCAATGGCTTTATAAAACAAGGATG** | **350** |
| **86** | **277706** | **277706** | **SNP1AA** | **H** | **2** | **N23L_277706** | **CCAATGGCTTTATAAAACAAGGATA** | **350** |
| **87** | **277706** | **277706** | **SNP1AA** | **H** | **3** | **N8-23R_277706** | **CCTTAGATCGCAGCAAAATATCGTC** | **350** |
| **88** | **369775** | **369775** | **SNP1AA** | **H** | **4** | **N8L_369775** | **TAATATCGCCTTTTATCCATTGGCT** | **281** |
| **89** | **369775** | **369775** | **SNP1AA** | **H** | **5** | **N23L_369775** | **TAATATCGCCTTTTATCCATTGGCC** | **281** |
| **90** | **369775** | **369775** | **SNP1AA** | **H** | **6** | **N8-23R_369775** | **CAATAGCACGCACATGATTTTC** | **281** |
| **91** | **518892** | **518892** | **SNP1AA** | **H** | **7** | **N8L_518892** | **ATATGTTGGTGCAGCTGATATGGAA** | **489** |
| **92** | **518892** | **518892** | **SNP1AA** | **H** | **8** | **N23L_518892** | **ATATGTTGGTGCAGCTGATATGGAG** | **489** |
| **93** | **518892** | **518892** | **SNP1AA** | **H** | **9** | **N8-23R_518892** | **CCCTGCTGCTGGATAAACCAC** | **489** |
| **94** | **698893** | **698893** | **SNP1AA** | **H** | **10** | **N8L_698893** | **TTGAAACATGGTAGCCTAAATCAAA** | **473** |
| **95** | **698893** | **698893** | **SNP1AA** | **H** | **11** | **N23L_698893** | **TTGAAACATGGTAGCCTAAATCAAG** | **473** |
| **96** | **698893** | **698893** | **SNP1AA** | **H** | **12** | **N8-23R_698893** | **AAAACCCATTGCAACAATATCAAC** | **473** |
