# Supplementary material for: Whole genome single nucleotide polymorphism based phylogeny of Francisella tularensis and its application to the development of a strain typing assay
Source: BMC Microbiol. 2009 Oct 7;9:213. doi: 10.1186/1471-2180-9-213 (PMC2767358; doi:10.1186/1471-2180-9-213)
Supplement: Additional file 3 — Whole genome resequencing call rates and SNPs for F. tularensis strains [file 1471-2180-9-213-S3.DOC]

**Additional File 3: Whole genome resequencing call rates and number of SNPs for *F. tularensis*** strains

| **S. No.** | **Isolate** | **Clade** | **Av. Filtered Call Rate (%)** | **Filtered SNPs** |
| --- | --- | --- | --- | --- |
| 1 | LVS | B | 96.034 | 15 |
| 2 | FRAN 004 | B | 95.563 | 16 |
| 3 | FRAN 025 | B | 94.543 | 497 |
| 4 | FRAN 029 | B | 95.442 | 532 |
| 5 | KY99-3387 | B | 97.585 | 534 |
| 6 | KY00-1708 | B | 95.622 | 576 |
| 7 | CA99-3992 | B | 96.136 | 588 |
| 8 | OR96-0463 | B | 96.503 | 590 |
| 9 | MO01-1673 | B | 96.013 | 592 |
| 10 | OR96-0246 | B | 97.920 | 594 |
| 11 | IN00-2758 | B | 96.481 | 600 |
| 12 | FRAN 012 | B | 95.787 | 605 |
| 13 | FRAN 024 | B | 94.036 | 2915 |
| 14 | UT02-1927 | A2 | 93.878 | 4732 |
| 15 | FRAN 028 | A2 | 92.433 | 4906 |
| 16 | WY96-3418 | A2 | 97.406 | 4948 |
| 17 | FRAN 027 | A2 | 93.084 | 5187 |
| 18 | FRAN 001 | A2 | 93.517 | 5335 |
| 19 | CA02-0099 | A2 | 96.059 | 5469 |
| 20 | MA00-2987 | A1 | 97.615 | 5929 |
| 21 | KS00-1817 | A1 | 95.484 | 6098 |
| 22 | AR01-1117 | A1 | 95.665 | 6103 |
| 23 | FRAN 030 | A1 | 94.616 | 6206 |
| 24 | SCHU S4 | A1 | 96.070 | 6295 |
| 25 | FRAN 031 | A1 | 94.995 | 6331 |
| 26 | FRAN 009 | A1 | 95.713 | 6351 |
| 27 | FRAN 006 | A1 | 95.962 | 6389 |
| 28 | FRAN 015 | A1 | 95.288 | 6405 |
| 29 | FRAN 032 | A1 | 95.599 | 6412 |
| 30 | FRAN 033 | A1 | 95.251 | 6416 |
| 31 | FRAN 023 | A1 | 95.691 | 6447 |
| 32 | FRAN 010 | A1 | 96.203 | 6450 |
| 33 | FRAN 005 | A1 | 96.301 | 6452 |
| 34 | FRAN 008 | A1 | 96.096 | 6452 |
| 35 | FRAN 014 | A1 | 95.829 | 6480 |
| 36 | FRAN 026 | A1 | 95.911 | 6484 |
| 37 | OK00-2732 | A1 | 96.491 | 6491 |
| 38 | FRAN 011 | A1 | 95.981 | 6517 |
| 39 | FRAN 007 | A1 | 96.673 | 6543 |
| 40 | FRAN 003 | *novicida* | 83.041 | 12407 |
