# Supplementary material for: Whole genome single nucleotide polymorphism based phylogeny of Francisella tularensis and its application to the development of a strain typing assay
Source: BMC Microbiol. 2009 Oct 7;9:213. doi: 10.1186/1471-2180-9-213 (PMC2767358; doi:10.1186/1471-2180-9-213)
Supplement: Additional file 4 — Quantitative SNP differences between the major phylogenetic nodes in the cladogram [file 1471-2180-9-213-S4.DOC]

**Additional File 4**: SNP differences between the major phylogenetic nodes in the cladogram

| **Subspecies/clade/subclade/node** | **Subspecies/clade/subclade/node** | **Node Differentiating SNPs** | **Minimum SNP Separation** | **Maximum SNP Separation** |
| --- | --- | --- | --- | --- |
| **B/50** | **A/4** | **782** | **3216** | **6432** |
| **B/50** | **A1/5** | **2079** | **4359** | **6432** |
| **B/50** | **A2/39** | **1255** | **3216** | **5332** |
| **B*/51** | **A/4** | **1319** | **3216** | **6432** |
| **B*/51** | **A1/5** | **2792** | **4359** | **6432** |
| **B*/51** | **A2/39** | **1886** | **3216** | **5332** |
| **B1/52** | **A/4** | **1448** | **3216** | **6416** |
| **B1/52** | **A1/5** | **2976** | **4359** | **6416** |
| **B1/52** | **A2/39** | **2043** | **3216** | **5300** |
| **B2/64** | **A/4** | **2200** | **4147** | **6432** |
| **B2/64** | **A1/5** | **4025** | **5330** | **6432** |
| **B2/64** | **A2/39** | **2899** | **4147** | **5332** |
| **B1/52** | **B2/64** | **135** | **453** | **602** |
| **A1/5** | **A2/39** | **1558** | **2229** | **2779** |
| **A1a/8** | **A1b/23** | **15** | **55** | **86** |

Node differentiating SNPs are locations at which all members of the first node share a common base call and all members of the second node share a different common base call. Minimum SNP separation is the number of SNP differences separating the two closest member strains representing each node. Maximum SNP separation is the number of SNP differences separating the two most distant members, each representing a node being compared.
