# Supplementary material for: Whole genome single nucleotide polymorphism based phylogeny of Francisella tularensis and its application to the development of a strain typing assay
Source: BMC Microbiol. 2009 Oct 7;9:213. doi: 10.1186/1471-2180-9-213 (PMC2767358; doi:10.1186/1471-2180-9-213)
Supplement: Additional file 5 — Features of in silico identified SNP diagnostic markers. [file 1471-2180-9-213-S5.DOC]

**Additional File 5: Features of *in silico* identified SNP diagnostic markers**

| **S. No.** | **SNP position^a^** | **Marker position^b^** | **Discriminating nodes^†^** | **Discriminating base** | | **Locus name** | **Gene symbol** | **Product^d^** | **Role category^e^** |
| --- | --- | --- | --- | --- | --- | --- | --- | --- | --- |
| **1** | **82272** | **82272** | **N5-N39** | **A** | **G** | **FTL_0087** |  | **Acetyltransferase protein** | **Unknown function** |
| **2** | **211514** | **211513** | **N4-N50** | **T** | **C** | **FTL_0210** | **valS** | **Valyl-tRNA synthetase** | **Protein synthesis** |
| **3** | **277706** | **277706** | **N8-N23** | **G** | **A** | **FTL_0292** |  | **Conserved hypothetical protein, pseudogene** | **Hypothetical protein** |
| **4** | **299153** | **299153** | **N52-N64** | **T** | **G** | **FTL_0310** | **aceF** | **Pyruvate dehydrogenase, E2 component** | **Energy metabolism** |
| **5** | **369775** | **369775** | **N8-N23** | **T** | **C** | **FTL_0399** | **purK** | **Phosphoribosylaminoimidazole carboxylase, ATPase subunit** | **Purines, pyrimidines, nucleosides, and nucleotides** |
| **6** | **470635** | **470635** | **N52-N64** | **G** | **A** | **FTL_0487** | **glgP^c^** | **Maltodextrin phosphorylase** | **Energy metabolism** |
| **7** | **482885** | **482885** | **N4-N50** | **T** | **C** | **FTL_0498** | **thrC** | **Threonine synthase** | **Amino acid biosynthesis** |
| **8** | **518892** | **518892** | **N8-N23** | **A** | **G** | **FTL_0535** |  | **Outer membrane protein** | **Unknown function** |
| **9** | **521982** | **521982** | **N5-N39** | **T** | **C** | **FTL_0538** | **fabZ** | **(3R)-Hydroxymyristoyl-(acyl-carrier protein) dehydratase** | **Fatty acid and phospholipid metabolism** |
| **10** | **530990** | **530990** | **N52-N64** | **G** | **A** | **Intergenic (FTL_0548- FTL_0547)** |  | **Intergenic** | **Intergenic** |
| **11** | **587086** | **587086** | **N4-N50** | **C** | **T** | **FTL_0599** | **wbtG^c^** | **Glycosyl transferase** | **Unknown function** |
| **12** | **684048** | **684048** | **N4-N50** | **G** | **A** | **FTL_0696** |  | **ABC transporter, ATP-binding protein** | **Transport and binding proteins** |
| **13** | **698893** | **698893** | **N8-N23** | **A** | **G** | **FTL_0708** |  | **Hypothetical protein** | **Hypothetical protein** |
| **14** | **726518** | **726518** | **N52-N64** | **A** | **C** | **FTL_0737** |  | **Hypothetical membrane protein** | **Cell envelope** |
| **15** | **823355** | **823355** | **N52-N64** | **C** | **T** | **FTL_0841** |  | **Hypothetical lipoprotein** | **Hypothetical protein** |
| **16** | **917759** | **917759** | **N4-N50** | **C** | **A** | **FTL_0948** |  | **Conserved hypothetical protein** | **Hypothetical protein** |
| **17** | **956165** | **956165** | **N52-N64** | **C** | **T** | **FTL_0986** | **nrdA^c^** | **Ribonucleoside-diphosphate reductase, alpha subunit** | **Purines, pyrimidines, nucleosides, and nucleotides** |
| **18** | **1011425** | **1011423** | **N52-N64** | **A** | **G** | **FTL_1057** |  | **Conserved hypothetical membrane protein,pseudogene** | **Hypothetical protein** |
| **19** | **1014623** | **1014621** | **N4-N50** | **T** | **C** | **FTL_1060** |  | **D-alanyl-D-alanine carboxypeptidase (Penicillin binding protein) family protein** | **Unknown function** |
| **20** | **1025460** | **1025458** | **N5-N39** | **T** | **C** | **FTL_1072** | **dxs** | **1-deoxy-D-xylulose 5-phosphate synthase** | **Biosynthesis of cofactors, prosthetic groups, and carriers** |
| **21** | **1083612** | **1083610** | **N52-N64** | **A** | **G** | **FTL_1141** | **fabH^c^** | **3-oxoacyl-[acyl carrier protein] synthase III** | **Fatty acid and phospholipid metabolism** |
| **22** | **1136971** | **1136969** | **N4-N50** | **T** | **C** | **FTL_1188** |  | **Conserved hypothetical protein** | **Hypothetical protein** |
| **23** | **1401061** | **1401057** | **N5-N39** | **G** | **A** | **FTL_1476** | **pgi** | **Glucose-6-phosphate isomerase** | **Energy metabolism** |
| **24** | **1413068** | **1413064** | **N5-N39** | **C** | **T** | **FTL_1487** |  | **Uridine phosphorylase** | **Purines, pyrimidines, nucleosides, and nucleotides** |
| **25** | **1476018** | **1476016** | **N5-N39** | **A** | **G** | **FTL_1546** |  | **Pyridoxine/pyridoxal 5-phosphate biosynthesis protein** | **Biosynthesis of cofactors, prosthetic groups, and carriers** |
| **26** | **1482239** | **1482237** | **N5-N39** | **T** | **G** | **Intergenic (FTL_1550-FTL_1551)** |  | **Intergenic** | **Intergenic** |
| **27** | **1507435** | **1507433** | **N5-N39** | **A** | **C** | **FTL_1580** | **rsgA** | **Conserved hypothetical protein** | **Protein synthesis** |
| **28** | **1555228** | **1555226** | **N5-N39** | **C** | **T** | **FTL_1624** |  | **Major facilitator superfamily (MFS) transport protein** | **Transport and binding proteins** |
| **29** | **1574929** | **1574927** | **N8-N23** | **G** | **C** | **FTL_1644** | **glpK** | **Glycerol kinase** | **Energy metabolism** |
| **30** | **1581977** | **1581975** | **N4-N50** | **T** | **C** | **FTL_1653** |  | **Peptide transport system substrate-binding protein** | **Transport and binding proteins** |
| **31** | **1659094** | **1659091** | **N52-N64** | **C** | **T** | **FTL_1726** | **parE** | **Topoisomerase IV, subunit B** | **DNA metabolism** |
| **32** | **1802570** | **1802567** | **N4-N50** | **G** | **A** | **FTL_1870** |  | **Putative ABC transporter ATP-binding protein** | **Transport and binding proteins** |

a: SNP position based on the LVS reference genome tiled on the array

b: SNP marker position in Genbank LVS genome, Accession: AM 233362

c: Biohealthbase based assignment (www.biohealthbase.org). All the other gene symbols were common to both TIGR and biohealthbase databases

d: Based on Genbank annotation

e: Based on CMR role category (<http://cmr.jcvi.org/cgi-bin/CMR/GenomePage.cgi?org=ntft11>)

†: N4 vs N50 for type A vs type B; N5 vs N39 for A1 vs A2; N8 vs N23 for A1a vs A1b and N52 vs N64 for B1 vs B2

Loci in red are on the forward strand and in blue are on the reverse strand
